# Albuminuria testing and nephrology care among insured US adults with chronic kidney disease: a missed opportunity

**DOI:** 10.1186/s12875-022-01910-9

**Published:** 2022-11-24

**Authors:** Chi D. Chu, Neil R. Powe, Michael G. Shlipak, Rebecca Scherzer, Sri Lekha Tummalapalli, Michelle M. Estrella, Delphine S. Tuot

**Affiliations:** 1 Department of Medicine, University of California, San Francisco, CA USA; 2 Department of Medicine, Priscilla Chan and Mark Zuckerberg San Francisco General Hospital, San Francisco, CA USA; 3 Kidney Health Research Collaborative, Department of Medicine, University of California, San Francisco, CA and San Francisco VA Health Care System, San Francisco, CA USA; 4 Division of Nephrology, Zuckerberg San Francisco General Hospital, 1001 Potrero Ave, Building 100, Room 342, San Francisco, CA 94110 USA; 5 Division of Healthcare Delivery Science & Innovation, Department of Population Health Sciences, Weill Cornell Medicine, New York, NY USA

**Keywords:** Chronic kidney disease, Nephrology referral, Albuminuria

## Abstract

**Background:**

In chronic kidney disease (CKD), assessment of both estimated glomerular filtration rate (eGFR) and albuminuria are necessary for stratifying risk and determining the need for nephrology referral. The Kidney Disease: Improving Global Outcomes clinical practice guidelines for CKD recommend nephrology referral for eGFR < 30 ml/min/1.73m^2^ or for urinary albumin/creatinine ratio ≥ 300 mg/g.

**Methods:**

Using a national claims database of US patients covered by commercial insurance or Medicare Advantage, we identified patients with CKD who were actively followed in primary care. We examined receipt of nephrology care within 1 year among these patients according to their stage of CKD, classified using eGFR and albuminuria categories. Multivariable logistic regression was used to examine odds of receiving nephrology care by CKD category, adjusting for age, sex, race/ethnicity, diabetes, heart failure, and coronary artery disease.

**Results:**

Among 291,155 patients with CKD, 55% who met guideline-recommended referral criteria had seen a nephrologist. Receipt of guideline-recommended nephrology care was higher among those with eGFR < 30 (64%; 11,330/17738) compared with UACR ≥300 mg/g (51%; 8789/17290). 59% did not have albuminuria testing. Those patients without albuminuria testing had substantially lower adjusted odds of recommended nephrology care (aOR 0.47 [0.43, 0.52] for eGFR < 30 ml/min/1.73m^2^). Similar patterns were observed in analyses stratified by diabetes status.

**Conclusions:**

Only half of patients meeting laboratory criteria for nephrology referral were seen by a nephrologist. Underutilization of albuminuria testing may be a barrier to identifying primary care patients at elevated kidney failure risk who may warrant nephrology referral.

**Supplementary Information:**

The online version contains supplementary material available at 10.1186/s12875-022-01910-9.

## Background

The heterogeneity in risk for kidney disease progression makes effective CKD risk stratification with assessment of both estimated glomerular filtration rate (eGFR) and albuminuria crucial [1]. Measurement of both eGFR and albuminuria are necessary for effective risk stratification [2–4]. Accordingly, the Kidney Disease: Improving Global Outcomes (KDIGO) clinical practice guidelines stage CKD severity by categories of eGFR (≥60, 45-59, 30-44, and < 30 ml/min/1.73m^2^) and albuminuria (urine albumin/creatinine ratio [UACR] < 30, 30-299, and ≥ 300 mg/g). Nephrology referral is recommended for patients with eGFR < 30 ml/min/1.73m^2^ and/or UACR ≥300 mg/g [5]. Prescription of some medication classes that decrease risk of kidney failure is recommended for individuals with UACR > 30 mg/g, including renin-angiotensin system blockers, sodium-glucose cotransporter 2 (SGLT2) inhibitors, and nonsteroidal mineralocorticoid antagonists in patients with diabetes [6–10].

Despite its critical role in risk stratification and management, albuminuria testing remains widely underutilized [11–13]. Importantly, the underutilization of albuminuria testing may hamper identification of high-risk persons with CKD who may benefit from nephrology care. Timely referral to nephrology care may allow for more aggressive management to prevent CKD progression and is associated with several clinical benefits, including improved vascular access planning, reduced hospitalizations, and greater likelihood of initiating home dialysis [14, 15]. This study aimed to examine receipt of nephrologist care by eGFR and albuminuria categories in a large population of US adults with CKD actively followed in primary care, with a focus on the association between albuminuria testing and likelihood of receiving nephrology care.

## Methods

We performed a cross-sectional analysis using the Optum Labs Data Warehouse, which includes deidentified claims and laboratory results from commercially insured and Medicare Advantage enrollees throughout the US. We assembled a study population of adults age ≥ 18 who had at least two primary care visits from January 1, 2015 to December 31, 2019 with laboratory evidence of CKD. CKD was defined by two outpatient eGFR values < 60 ml/min/1.73m^2^ separated by ≥90 days or two outpatient UACR values ≥30 mg/g separated by ≥90 days [5]. We applied the 2021 CKD-Epidemiology Collaboration equation to calculate eGFR because it is now the recommended by the joint task force of the American Society of Nephrology and National Kidney Foundation, and although not contemporary to the study period, its use establishes a baseline pattern of health care use for future comparison [16]. Because urine protein/creatinine ratio (UPCR) is frequently obtained as an alternative to UACR, we estimated additional UACR results using a validated conversion from UPCR [17]. The date of the second qualifying eGFR or UACR defined the index date for each patient. We excluded patients who previously received dialysis or kidney transplantation.

We determined the proportion of patients receiving nephrology care, defined as having at least one outpatient nephrology encounter within 12 months following the index date, according to KDIGO-based CKD categories. We used multivariable logistic regression to examine associations between albuminuria category and nephrology care, stratified by eGFR category, adjusting for age, sex, race/ethnicity, diabetes, heart failure, and coronary artery disease.

## Results

Our study population included 291,155 patients (mean age 72 ± 10 years; 58% female) with CKD. Table 1 describes characteristics of the study population.

**Table 1:** Study population characteristics by albuminuria category (*N* = 291,155)

Characteristic	Albuminuria Category			
	0-29 mg/g	30-299 mg/g	≥300 mg/g	Missing
n (%)	50,336 (17.3)	52,543 (18.0)	17,290 (5.9)	170,986 (58.7)
Age, mean (SD)	71.6 (8.8)	68.8 (11.2)	66.7 (12.2)	73.1 (9.2)
Female, n (%)	29,713 (59.0)	25,559 (48.6)	7280 (42.1)	105,824 (61.9)
Race and Ethnicity				
Asian	2313 (4.6)	4368 (8.3)	1333 (7.7)	5730 (3.4)
Black	12,574 (25.0)	10,718 (20.4)	4203 (24.3)	41,189 (24.1)
Hispanic	7138 (14.2)	10,450 (19.9)	3439 (19.9)	13,902 (8.1)
White	23,866 (47.4)	22,135 (42.1)	6740 (39.0)	93,078 (54.4)
Unknown	4445 (8.8)	4872 (9.3)	1575 (9.1)	17,087 (10.0)
Hypertension, n (%)	48,042 (95.4)	49,902 (95.0)	16,896 (97.7)	157,192 (91.9)
Diabetes mellitus, n (%)	27,946 (55.5)	37,163 (70.7)	12,875 (74.5)	46,563 (27.2)
HbA1c (%), mean (SD)	6.6 (1.3)	7.2 (1.7)	7.6 (1.9)	6.4 (1.3)
eGFR (ml/min/1.73m^2^), mean (SD)	49 (9)	67 (24)	53 (26)	49 (10)
eGFR category (ml/min/1.73m^2^), n (%)				
≥60	0 (0.0)	27,332 (52.0)	5377 (31.1)	0 (0.0)
45-59	37,136 (73.8)	14,967 (28.5)	4608 (26.7)	124,628 (72.9)
30-44	10,937 (21.7)	7550 (14.4)	3967 (22.9)	36,915 (21.6)
< 30	2263 (4.5)	2694 (5.1)	3338 (19.3)	9443 (5.5)
UACR (mg/g), median [IQR]	7 (3, 14)	66 (43, 118)	756 (451, 1516)	N/A
Congestive heart failure, n (%)	10,306 (20.5)	10,858 (20.7)	5288 (30.6)	40,099 (23.5)
Coronary artery disease, n (%)	4779 (9.5)	5320 (10.1)	2403 (13.9)	17,759 (10.4)

All comparisons have *p* < 0.001.

*Abbreviations: BP* blood pressure, *eGFR* estimated glomerular filtration rate, *HbA1c* hemoglobin A1c, *IQR* interquartile range, *SD* standard deviation, *UACR* urine albumin/creatinine ratio

Overall, 59% (*n* = 170,986) did not have albuminuria testing available. Missing albuminuria was positively associated with older age, lower prevalence of hypertension and diabetes, and higher eGFR: 53%, 62%, and 69% missing for eGFR of < 30, 30-44, and 45-59 ml/min/1.73m^2^ respectively.

**Fig. 1:**
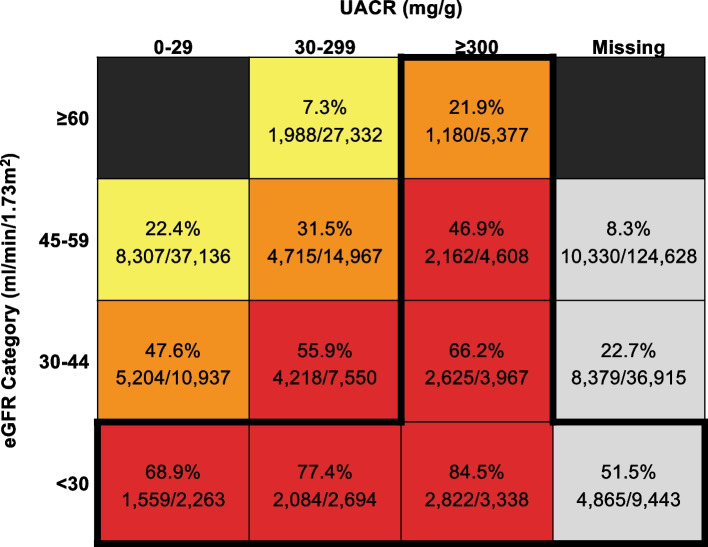
Proportion of Patients Receiving Nephrology Care by eGFR and Albuminuria Category. The intensity of coloring (yellow, orange, red) represents the risk for CKD progression and kidney failure based on the KDIGO classification by eGFR and UACR; patients with missing UACR (gray colored boxes) are not assigned a risk level by KDIGO. The bold outline represents categories for which nephrology referral is guideline-recommended. Abbreviations: CKD = chronic kidney disease; eGFR = estimated glomerular filtration rate; KDIGO = Kidney Disease: Improving Global Outcomes; UACR = urine albumin/creatinine ratio

Among all patients with CKD, 21% (*n* = 60,438) received nephrology care. Figure 1 shows the proportion receiving nephrology care by CKD category. Among the 31,690 patients within CKD categories of guideline-recommended referral, only 55% (*n* = 17,297) received nephrology care. Receipt of guideline-recommended nephrology care was higher among those with eGFR < 30 (11,330/17,738, 64%) compared with UACR ≥300 (8789/17,290, 51%). Within every eGFR category, patients with missing albuminuria were least likely to receive nephrology care.

**Fig. 2:**
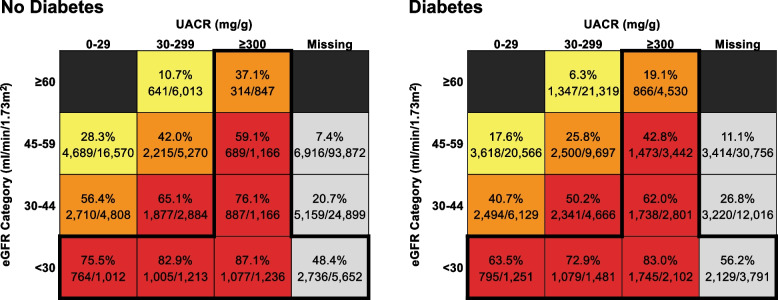
Proportion of Patients Receiving Nephrology Care Stratified by eGFR, Albuminuria, and Diabetes Status. The intensity of coloring (yellow, orange, red) represents the risk for CKD progression and kidney failure based on the KDIGO classification by eGFR and UACR; patients with missing UACR (gray colored boxes) are not assigned a risk level by KDIGO. The bold outline represents categories for which nephrology referral is guideline-recommended. Abbreviations: CKD = chronic kidney disease; eGFR = estimated glomerular filtration rate; KDIGO = Kidney Disease: Improving Global Outcomes; UACR = urine albumin/creatinine ratio

When stratified by diabetes status, we found that 25% (*n* = 42,185/166,608) of patients without diabetes had albuminuria testing, compared with 63% (*n* = 77,984/124,547) among patients with diabetes. The proportion of patients receiving nephrology care was higher in more severe eGFR and albuminuria categories for both patients with and without diabetes (Fig. 2). Prevalence of guideline-recommended nephrology referral was 61% (7472/12,292) among patients without diabetes, compared with 51% (9825/19,398) among patients with diabetes.

With respect to CVD, the proportion of patients with available albuminuria testing was similar in patients without CVD (42%) and patients with CVD (40%). The proportion of patients receiving nephrology care by CVD status is shown in Fig. S1. Guideline-recommended nephrology care was 51% (9722/18,921) among patients without CVD, compared with 61% (7575/12,369) among patients with CVD.

**Fig. 3:**
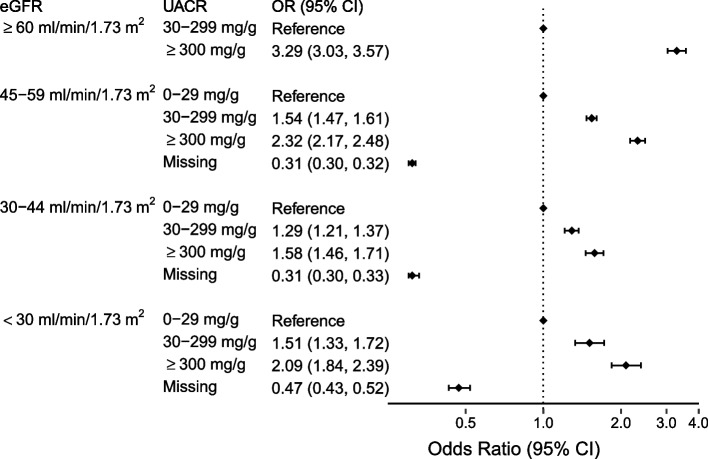
Odds Ratios and 95% Confidence Intervals for Nephrology Care by Albuminuria Category Stratified by eGFR Category. Odds ratios are adjusted for age, sex, race/ethnicity, diabetes, heart failure, and coronary artery disease. Abbreviations: CI = confidence interval; eGFR = estimated glomerular filtration rate; OR = odds ratio; UACR = urine albumin/creatinine ratio

In multivariable-adjusted models, more severe albuminuria was consistently associated with higher odds of nephrology care within each given category of eGFR (Fig. 3). Missing albuminuria was consistently associated with lower odds of nephrology care.

## Discussion

In a national cohort of adults with CKD, only half of patients meeting guideline-recommended referral criteria based on eGFR and albuminuria were seen by a nephrologist. More severe albuminuria was associated with greater likelihood of receiving nephrology care. However, over half of patients were missing albuminuria measures; these patients were substantially less likely to receive nephrology care for any given eGFR category. Because this study was limited to a population with consistent access to care based on continuous enrollment in insurance with primary care visits, rates of recommended nephrology care may be even lower in other settings.

Our results showing low UACR testing among patients with laboratory evidence of CKD complement prior work by Alfego et al. finding widespread UACR underutilization among patients at risk for CKD, i.e., those with hypertension or diabetes [11]. In addition, Alfego et al. found low rates of CKD diagnosis, even among patients whose testing confirmed CKD in a high-risk KDIGO category. The present study identified care gaps extending beyond underdiagnosis, as we found many patients with high-risk CKD did not receive guideline-recommended nephrology care. Together, these findings underscore the need for increased awareness of the indications for UACR testing as well as identification of CKD and appropriate referral based on the test results.

Reasons for low UACR testing are likely multifactorial. Higher UACR testing rates among patients with diabetes compared to those without diabetes has been consistently documented [11, 12], and may relate to national quality metrics and clinical practice guidelines from the American Diabetes Association which recommend annual UACR testing for patients with diabetes [18, 19]. In contrast, recommendations for UACR testing among patients with hypertension have been less consistent. The 2017 American College of Cardiology/American Heart Association hypertension guidelines include UACR in a list of “optional” testing; however, in the same guideline, the choice of antihypertensive therapy depends on presence/absence of albuminuria [20]. Since these guidelines were published, the availability of therapies, such as SGLT2 inhibitors that have shown overwhelming kidney and cardiovascular benefits in albuminuric CKD, has made UACR testing even more imperative irrespective of diabetes status [9]. Detection of albuminuria by UACR testing affords early detection of CKD and thus early initiation of these therapies, when their preventive benefit can be maximized. Of note, the majority of patients with CKD solely defined by albuminuria category are managed in the primary care setting with the dual goal of optimizing therapy to prevent CVD and CKD progression. Consequently, increasing primary care awareness of the prognostic and therapeutic implications of UACR testing is essential for optimal CKD care and preventing adverse cardiorenal outcomes.

Efforts to improve awareness and evidence-based care delivery for CKD are underway. In the US, the Advancing American Kidney Health Executive Order outlined goals for prevention, detection, and treatment of CKD in addition to a CKD awareness campaign to improve public knowledge of CKD and its risk factors [21]. There is also increasing recognition of a role for well-designed quality metrics relevant to CKD care, as most existing metrics for nephrology relate to dialysis care [22, 23]. Updated clinical practice guidelines may also increase awareness of the need for UACR testing. For example, the 2021 National Institute for Health and Care Excellence (NICE) CKD guideline recommends risk-based nephrology referral using the Kidney Failure Risk Equation (KFRE), a prediction model that requires both eGFR and UACR as input variables [1, 24, 25]. A study of current practice in the US examining KFRE-predicted risk and nephrology care found nearly half of patients with identifiably high kidney failure risk had not been seen by a nephrologist [26]. However, in that study, the KFRE could not be calculated in nearly 75% of patients with CKD due to missing UACR. Thus, strategies to improve UACR testing among at-risk patients are also needed to facilitate health services research and care delivery surveillance efforts.

Strengths of our study include the large, multi-year population of patients with CKD in primary care from across the US. The use of claims rather than electronic health record data allows capture of nephrology encounters across different health systems. Limitations include our inability to identify referrals to nephrology that were requested but had not yet occurred. Generalizability of commercial and Medicare Advantage data to other populations may be limited. We used the 2021 CKD-EPI equation for eGFR, which does not necessarily reflect eGFR values available to clinicians during the study period, when both the 2009 CKD-EPI and Modification of Diet in Renal Disease equations were in widespread use by different laboratories [27]. Causal relationships between albuminuria and nephrology care cannot be ascertained due to the observational design.

## Conclusions

In a large population of primary care patients with CKD, only half of patients meeting laboratory criteria for nephrology referral were seen by a nephrologist. Underutilization of albuminuria testing may be a barrier to identifying primary care patients at elevated kidney failure risk who may warrant nephrology referral.

## Supplementary Information

**Additional file 1.** Supplementary Material: Albuminuria Testing and Nephrology Care among Insured US Adults with Chronic Kidney Disease: A Missed Opportunity. Table S1. Diagnosis and Procedure Codes for Comorbidities. Fig. S1. Proportion of Patients Receiving Nephrology Care Stratified by eGFR, Albuminuria, and CVD Status

## Data Availability

The data that support the findings of this study are available from Optum Labs but restrictions apply to the availability of these data, which were used under license for the current study, and so are not publicly available. Data are however available from the authors upon reasonable request and with permission of Optum Labs.

## Abbreviations

CKD: chronic kidney disease
CKD-EPI: Chronic Kidney Disease Epidemiology Collaboration
CVD: cardiovascular disease
eGFR: estimated glomerular filtration rate
KDIGO: Kidney Disease: Improving Global Outcomes
KFRE: Kidney Failure Risk Equation
SGLT2: sodium-glucose cotransporter 2
UACR: urine albumin/creatinine ratio
UPCR: Surine protein/creatinine ratio
