# Supplementary material for: Albuminuria testing and nephrology care among insured US adults with chronic kidney disease: a missed opportunity
Source: BMC Prim Care. 2022 Nov 24;23:299. doi: 10.1186/s12875-022-01910-9 (PMC9700954; doi:10.1186/s12875-022-01910-9)
Supplement: Supplementary file 1 — Additional file 1. Supplementary Material: Albuminuria Testing and Nephrology Care among Insured US Adults with Chronic Kidney Disease: A Missed Opportunity. Table S1. Diagnosis and Procedure Codes for Comorbidities. Fig. S1. Proportion of Patients Receiving Nephrology Care Stratified by eGFR, Albuminuria, and CVD Status [file 12875_2022_1910_MOESM1_ESM.docx]

**Supplementary Material: Albuminuria Testing and Nephrology Care among Insured US Adults with Chronic Kidney Disease: A Missed Opportunity**

Chi D. Chu MD MAS^1,2,3^, Neil R. Powe MD MPH MBA^1,2^, Michael G. Shlipak MD MPH^1,3^, Rebecca Scherzer PhD^3^, Sri Lekha Tummalapalli MD MAS^3,4^, Michelle M. Estrella MD MHS^1,3^, Delphine S. Tuot MDCM MAS^1,2^

^1^ Department of Medicine, University of California, San Francisco, CA

^2^ Department of Medicine, Priscilla Chan and Mark Zuckerberg San Francisco General Hospital, San Francisco, CA

^3^ Kidney Health Research Collaborative, Department of Medicine, University of California, San Francisco, CA and San Francisco VA Health Care System, San Francisco, CA

^4^ Division of Healthcare Delivery Science & Innovation, Department of Population Health Sciences, Weill Cornell Medicine, New York, NY

**Supplementary Material Contents**

Table S1. Diagnosis and Procedure Codes for Comorbidities

Figure S1. Proportion of Patients Receiving Nephrology Care Stratified by eGFR, Albuminuria, and CVD Status

**Table S1. Diagnosis and Procedure Codes for Comorbidities**

| **Condition** | **Codes** |
| --- | --- |
| End-Stage Kidney Disease | CPT-4: 90935, 90937, 90945, 90947, 99512  ICD-9: 403.01, 403.11, 403.91, 404.02, 404.03, 404.12, 404.13, 404.92, 404,93, 585.5, 585.6, V45.11  ICD-10: I12.0, I13.11, I13.2, N18.5, N18.6 |
| Diabetes | ICD-9: 250, 790.2, 790.21, 790.22, 790.29, 648.8x, 648.0x, 791.5, 277.7  ICD-10: E08x, E09x, E10x, E11x, E13x |
| Coronary Heart Disease | ICD-9: 410.x, 412.x  ICD-10: I21.x, I22.x, I25.2 |
| Heart Failure | ICD-9: 398.91, 402.01, 402.11, 402.91, 404.01, 404.03, 404.11, 404.13, 404.91, 404.93, 428.x  ICD-10: I09.81, I11.0, I13.0, I13.2, I50 |
| Cerebrovascular Disease | ICD-9: 430.x, 431.x, 432.x, 433.x1, 434.x1, 435.x, 436.x, 438.x  ICD-10: V12.54, G45.0, G45.1, G45.2, G45.8, G45.9, G46.x, I60.x, I61.x, I62.x, I63.x, I69.x, Z86.73 |

Abbreviations: CPT = Current Procedural Terminology, ICD = International Classification of Disease.

Figure S1. Proportion of Patients Receiving Nephrology Care Stratified by eGFR, Albuminuria, and CVD Status

The intensity of coloring (yellow, orange, red) represents the risk for CKD progression and kidney failure based on the KDIGO classification by eGFR and UACR; patients with missing UACR (gray colored boxes) are not assigned a risk level by KDIGO. The bold outline represents categories for which nephrology referral is guideline-recommended. CVD is defined as a composite of heart failure and coronary artery disease. Abbreviations: CKD = chronic kidney disease; CVD = cardiovascular disease; eGFR = estimated glomerular filtration rate; KDIGO = Kidney Disease: Improving Global Outcomes; UACR = urine albumin/creatinine ratio.
